# Management of major bile duct injury after laparoscopic cholecystectomy: a case report

**DOI:** 10.1186/1752-1947-3-44

**Published:** 2009-01-31

**Authors:** Andreas Manouras, Nikolaos Pararas, Pantelis Antonakis, Emannuel E Lagoudiannakis, George Papageorgiou, Ioannis G Dalianoudis, Manoussos M Konstadoulakis

**Affiliations:** 1 1st Department of Propaedeutic Surgery, Hippocrateion Hospital, Athens Medical School, University of Athens, Athens, Greece; 2 Second Department of Surgery, 417NIMTS, Athens, Greece; 3 Endoscopic Radiology Department, Naval Hospital of Athens, Athens, Greece

## Abstract

**Introduction:**

Bile duct injury is a severe and potentially life-threatening complication of laparoscopic cholecystectomy. Several series have described a 0.5% to 0.6% incidence of bile duct injury during laparoscopic cholecystectomy. The aim of this study was to analyze the presentation, characteristics, related investigation, and treatment results of major bile duct injuries after laparoscopic cholecystectomy.

**Case presentation:**

A rare case of a 48-year-old Greek woman with a triple bile duct injury (right and left hepatic duct ligation and common bile duct cross-section) is presented. A Roux en Y hepaticojejunostomy was performed after repeated endoscopic retrograde cholangiopancreatographies, percutaneous transhepatic catheterization of the ducts and magnetic resonance cholangiographies to delineate the biliary anatomy and assess the level of injury.

**Conclusion:**

Early recognition and an adequate multidisciplinary approach are the cornerstones for the optimal final outcome. Suboptimal management of injuries often leads to more extensive damage to the biliary tree and its vasculature. Early referral to a tertiary care center with experienced hepatobiliary surgeons and skilled interventional radiologists would appear to be necessary to assure optimal results.

## Introduction

Gallstone disease is one of the most common digestive health problems [1]. Laparoscopic cholecystectomy (LC) is now the gold standard for gallbladder removal in the management of symptomatic cholelithiasis with decreased postoperative morbidity and mortality. Still, bile duct injuries are reported to be more severe and more common when compared to open cholecystectomy [2-5] with a reported incidence of up to 0.6% for laparoscopic versus 0.1% for open cholecystectomy [5]. These injuries are a disaster for both the patient and the surgeon because of the associated morbidity, prolonged hospitalization, and mortality [6].

The management of patients following major bile duct injury is a surgical challenge often requiring the skills of experienced hepatobiliary surgeons at tertiary referral centers [7]. Collaboration among surgeons, gastroenterologists and interventional radiologists is imperative in the care of such injuries.

The aim of this study was to analyze the presentation, characteristics, related investigation, and treatment results of a case with major complex bile duct injury after LC.

## Case presentation

A 48-year-old Greek female patient was referred to our institution for the management of biliary trauma after laparoscopic cholecystectomy. In her medical history, she had had two laparotomies: an ileoanal anastomosis with j pouch for ulcerative colitis 15 years ago, while 5 years ago she was driven to the operating room after a colonoscopy for peritonitis. Due to non-specific upper gastrointestinal symptoms she had had an upper abdominal ultrasound (US) that revealed cholelithiasis (at least two gallstones of diameter 0.8 and 0.7 cm) 2 months before surgery and elective laparoscopic cholecystectomy was performed 18 months before her referral to us.

**Figure 1:**
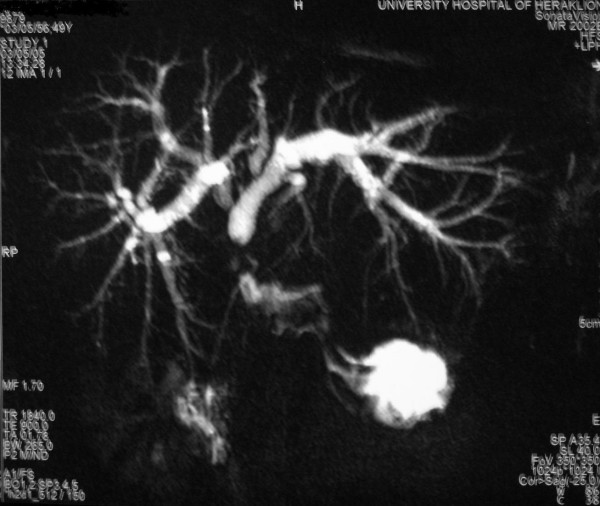
**A triple bile duct injury, with right and left hepatic duct ligation and common bile duct cross-section (Bismuth type V), was revealed in magnetic resonance imaging**.

**Figure 2:**
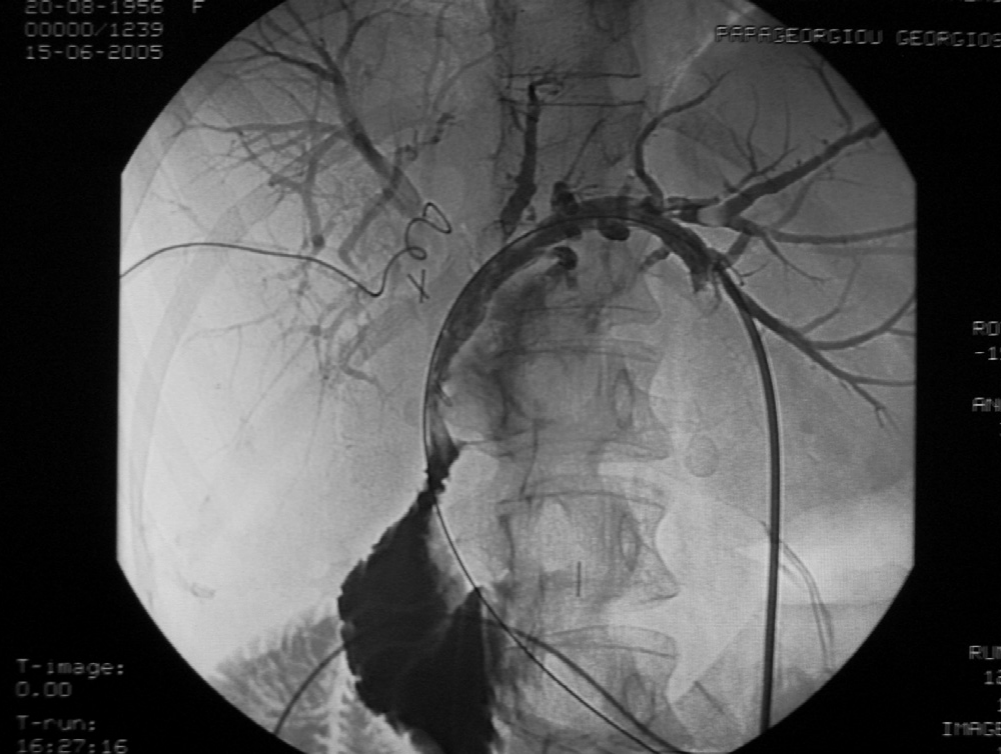
**Percutaneous decompression of both left and right biliary ducts was successful**. A false route was created to the duodenum through partial opening of the left duct clip from the guidewire.

The duration of laparoscopic cholecystectomy was 150 minutes while the procedure was completed laparoscopically and there is no record of intraoperatively identified biliary injury. From the 2nd postoperative day and up to her referral to our institution, recurrent episodes of cholangitis with severe pain, fever with chills and jaundice began. Magnetic resonance cholangiography (MRC) was performed in order to delineate the biliary anatomy and assess the level of injury. A triple bile duct injury, with right and left hepatic duct ligation and common bile duct cross-section, was revealed (Bismuth type V, Figure 1). Attempts at permanent biliary decompression with repeated endoscopic retrograde cholangiopancreatographies (ERCP), combined with percutaneous transhepatic duct catheterization failed and for 1 year postoperatively bile drained from abdominal drains. On the 13th postoperative month, she was referred to another hospital for biliary draining (Figure 2). Through the left drain, a guidewire was passed only to be found later during ERCP in the duodenum in a place other than the papilla of Vater via a false route. On the next episode of cholangitis, both left and right biliary trees were successfully decompressed and 18 months after LC, she was referred to our hospital for surgical reconstruction.

The intraoperative findings were as follows: the hepatic duct was cut and double ligated with clips, the right anterior hepatic duct was closed with an endo-clip, the right posterior hepatic duct was cut and ligated with one endo-clip, the left hepatic duct was cut and had a catheter passing through a partially open clip via a false route to the duodenum while the right hepatic artery was clip-ligated as well. After partial resection of segment IV of the liver, extensive dissection of the biliary tree was performed and then a plastic reconstruction of all major right hepatic ducts to a "common" right hepatic duct was performed with PDS 6-0. Finally, Roux en Y hepaticojejunostomy of both the newly formed common right hepatic duct and the left hepatic duct was performed at separate sites. The catheter in the left hepatic duct was left in place while the one in the right hepatic duct was removed. Three months after the operation, cholangiography showed patency of the left and right hepatic ducts (Figure 2) and after removal of the remaining stent, the patient has had no complaints. The liver function tests have so far returned results within the normal limits.

## Discussion

Biliary injuries include biliary leakage, hemobilia, and biliary fistula. The pattern of bile duct injuries has changed or become more complicated in recent years. There have been a few proposals to classify postoperative strictures and bile duct injuries. The Corlette-Bismuth classification (Table 1) is based on the length of the proximal biliary stump but not on the nature and length of the lesion. A detailed subdivision into minor and major bile duct injuries has been proposed by McMahon. Minor injuries include laceration of the cystic to common bile duct junction and laceration of the common hepatic duct over less than 25% of the duct diameter. Major injuries include laceration over more than 25% of the bile duct diameter, transection of the common hepatic or common bile duct, or the development of postoperative stricture. Another classification is by Strasberg (Table 2), and this is the most detailed classification as all types of injury, including leaks can be classified [3]. It is quite difficult to obtain the exact incidence rate of iatrogenic bile duct injuries because bile duct injuries can be attributable to the negligence of surgeons and are sometimes deliberately evaded in the hospital records, where these injuries are referred to as anatomical abnormalities or agenesis of the gallbladder [8].

**Table 1:** Corlette-Bismuth classification

Type 1	Low common hepatic duct stricture, with a length of the common hepatic duct stump of > 2 cm
Type 2	Middle stricture: length of common hepatic duct < 2 cm
Type 3	Hilar stricture, no remaining common hepatic duct, but the confluence is preserved
Type 4	Hilar stricture, with involvement of confluence and loss of communication between right and left hepatic duct
Type 5	Combined common hepatic and aberrant right hepatic duct injury, separating from the distal common bile duct

**Table 2:** Strasberg classification

Type A	Bile leak from cystic duct or liver bed without further injury
Type B	Partial occlusion of the biliary tree, most frequently of an aberrant right hepatic duct
Type C	Bile leak from duct (aberrant right hepatic duct) that is not communicating with the common bile duct
Type D	Lateral injury of biliary system, without loss of continuity
Type E	Circumferential injury of biliary tree with loss of continuity

The treatment of patients with major bile duct injury (MBDI) after LC is a difficult problem and depends on the time of diagnosis after the initial injury and the type, extent and level of the injury. The aim of the treatment is immediate management of the associated sepsis, fistula, and obstruction of the biliary system. Identification and categorization of the type of MBDI are the next steps. Once this is done, definitive repair of the injury should be performed. Postoperative follow-up and guidance, are vital parts of this prolonged treatment protocol. The reported incidence of MBDI after laparoscopic cholecystectomy has been shown to be higher than that after open cholecystectomy [9]. Several risk factors have been identified, mainly dangerous pathology, dangerous anatomy, and dangerous surgery [10]. In spite of the recognition of these well established risk factors, MBDI continues to be a problem in laparoscopic surgery. Furthermore, it may be missed during laparoscopic cholecystectomy [11].

During cholecystectomy, much emphasis is given to complete exposure of the operating area. During the exposure of peritoneal attachments in Calot's triangle, anatomical variations should be clearly identified, and the cystic duct should not be separated until the junction of the common hepatic and cystic ducts is positively identified. There is no confluence of any other abnormal ducts into the cystic duct.

Sometimes the anatomical structure of Calot's triangle is not very clear because of congestion, edema and fragility of the tissues around the cystic duct in acute suppurative or gangrenous cholecystitis. Fibrous tissue scars are often formed in Calot's triangle in atrophic cholecystitis. It is more difficult to avoid intraoperative bile duct injuries (IBDI) in such conditions, when correct identification of Calot's triangle is less likely.

Injuries to the bile duct system during laparoscopic cholecystectomy are an unaltered cause for concern not necessarily related to the "learning curve" of the operating surgeon as suggested in the past [12]. In recent studies, it was demonstrated that in more than one-third of all bile duct injuries, the basic cause of error is not the inexperience of the surgeon but the use of an improper approach to the fundamental structures of the extrahepatic biliary tree because of a visual perceptual illusion [12]. Correspondingly, in most cases, the problem is not recognized at the time of the initial procedure, particularly in the presence of acute inflammation or chronic fibrosis. The role of intraoperative cholangiography and laparoscopic ultrasonography in prevention of MBDI during laparoscopic cholecystectomy is a matter of ongoing debate [13].

**Figure 3:**
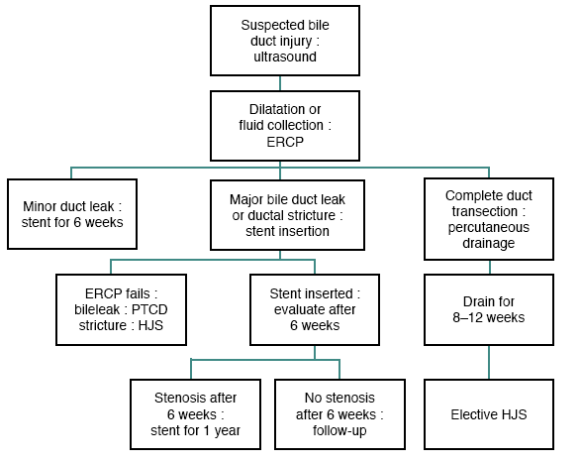
**Suggested flow diagram for patients with suspected bile duct injury after laparoscopic cholecystectomy **[3].

Proper diagnosis and appropriate treatment of MBDI, are paramount in preventing life-threatening complications of cholangitis, biliary cirrhosis, portal hypertension, end-stage liver disease, and death. At the time of referral, all patients with suspected bile duct injury should undergo US and computed tomography (CT) so that any dilatation or fluid collection can be found. Those techniques must be combined with magnetic resonance cholangiopancreatographies (MRCP), ERCP or percutaneous transhepatic catheterization (PTC) in order to delineate the biliary anatomy [3]. All patients who do not recover immediately after cholecystectomy by definition are candidates for having a bile duct injury. Those patients should follow the proposed flow diagram as depicted in Figure 3. Early referral to tertiary care centers with expertise in biliary surgery may limit further operations, complications, time to definitive repair, and mortality.

Pre-operative imaging studies such as magnetic resonance cholangiographies (MRC), ERCP, and PTC correctly delineate the location and nature of MBDI [3,14]. Surgery should only be contemplated when the patient is stabilized and the MBDI has been correctly classified. The success of the operating procedure depends directly on the proper and accurate delineation of the MBDI. If the injury is recognized in the early postoperative period (2 to 7 days), involves a relatively distal lesion below the bifurcation and is not associated with biliary leakage, abscess formation and sepsis, early reconstruction can be considered. When we have involvement of the bifurcation, percutaneous biliary drainage is preferred with elective repair after 6 to 8 weeks [3]. The control of sepsis and the ongoing bile leak are the primary goals of the initial management of a bile duct injury. If this can be accomplished, proceeding with surgical reconstruction is not urgent. In fact, reconstruction in the face of peritonitis portends a statistically poorer outcome in patients.

Once the sepsis and leaks have been controlled and the MBDI is classified, a hepaticojejunostomy should be constructed to a Roux-en-Y jejunal limb, or less commonly an end to side Roux-en-Y choledochojejunostomy. Satisfactory results have been reported by many authors using the Roux-en-Y hepaticojejunostomy. For strictures involving the bifurcation of left or right hepatic ducts, bilateral hepaticojejunostomies may be necessary. Level of injury is an important factor; the greater the level of the injury, the poorer the outcome after the procedure. Other factors include the timing of the repair, the performance of pre-operative cholangiography, the choice of surgical procedure, the expertise of the surgeon performing the repair, and the presence of concomitant vascular injury [11]. There are no data in the literature to show the exact incidence of recurrent stricture requiring revision hepaticojejunostomy after LC. In this situation, the level of the anastomosis is always greater than the original one. As reported in the literature, the outcome of surgical reconstruction mainly for major lesions or failure of endoscopic treatment is dependent on the timing of the reconstruction [3]. Postoperatively, as we can see from the literature, the transhepatic catheters should stay for external gravity drainage until day 5 when a cholangiogram should be performed. If no leaks or strictures are detected the transhepatic catheters should be capped (internalized). Follow-up cholangiograms should be obtained at 1 and 3 months postoperatively except if otherwise indicated. Catheters should be removed between 3 and 6 months postoperatively depending on the level of the injury and appearance of the cholangiogram [9]. After open cholecystectomy, recurrent biliary stricture has been observed in 10–30% of cases [15]. Moreover, patients with recurrent stricture are at higher risk of developing further restenosis. The number of previous surgical attempts also greatly influences the outcome. With repeated attempts to correct the failed repair, the stricture becomes ever greater, making the next repair even more difficult and the result even more unpredictable. To avoid these problems, it has been argued that patients with MBDI after LC should always be referred to a specialist center and that any attempts at repair outside tertiary units should be discouraged.

## Conclusion

In summary, MBDI after LC is a major problem that requires a multidisciplinary approach at a tertiary level center. Sepsis, biliary leaks, and collections should be managed appropriately, and proper classification of the MBDI via imaging needs to be done before the surgical repair. Roux-en-Y hepaticojejunostomy yields excellent results in these cases. Life-threatening complications can occur as a result of delayed referral or, rarely, after surgical repair. Although overall complications are frequent, almost all can be managed non-operatively. These data support the concept of early referral to a tertiary care center with experienced hepatobiliary surgeons and skilled interventional radiologists to assure optimal short-term and long-term outcomes.

## Abbreviations

CT: computed tomography; ERCP: endoscopic retrograde cholangiopancreatography; LC: laparoscopic cholecystectomy; PTC: percutaneous transhepatic cholangiography; US: ultrasound; IBDI: intraoperative bile duct injuries; MRC: magnetic resonance cholangiography; MBDI: major bile duct injuries

## Competing interests

The authors declare that they have no competing interests.

## Authors' contributions

<Author: From the BioMed guidelines, authors IGD, MMK and VK don't seem to qualify as authors. An "author" is generally considered to be someone who has made substantive intellectual contributions to a published study. To qualify as an author one should 1) have made substantial contributions to conception and design, or acquisition of data, or analysis and interpretation of data; 2) have been involved in drafting the manuscript or revising it critically for important intellectual content; and 3) have given final approval of the version to be published. Each author should have participated sufficiently in the work to take public responsibility for appropriate portions of the content. Acquisition of funding, collection of data, or general supervision of the research group, alone, does not justify authorship. All contributors who do not meet the criteria for authorship should be listed in an acknowledgments section. Examples of those who might be acknowledged include a person who provided purely technical help, writing assistance, or a department chair who provided only general support. After all authors meeting they have decided that IGD and MMK should be included to the authors as they have played an importaned role to the manuscript conception and they agree that VK should be listed in the acknowledgement section>

AM carried out the operation and contributed to acquisition of consent and critical review of the manuscript. NP, PA, EEL and GP all contributed to manuscript conception, research, acquisition of data, drafting and writing of the manuscript. IGD contributed to research, organizing, drafting, and writing of the manuscript. MMK contributed to writing of the manuscript. All authors read and approved the final manuscript.

## Consent

Written informed consent was obtained from the patient for publication of this case report and any accompanying images. A copy of the written consent is available for review by the Editor-in-Chief of this journal.
